# Pain, osteolysis, and periosteal reaction are associated with the STRYDE limb lengthening nail: a nationwide cross-sectional study

**DOI:** 10.1080/17453674.2021.1903278

**Published:** 2021-03-24

**Authors:** Jan Duedal Rölfing, Søren Kold, Tobias Nygaard, Mindaugas Mikuzis, Michael Brix, Christian Faergemann, Martin Gottliebsen, Michael Davidsen, Juozas Petruskevicius, Ulrik Kähler Olesen

**Affiliations:** a Orthopaedic Reconstruction and Children’s Orthopaedics, Aarhus University Hospital, Aarhus;; b Department of Clinical Medicine, Aarhus University, Aarhus;; c Department of Orthopaedics, Interdisciplinary Orthopaedics, Aalborg University Hospital, Aalborg;; d Department of Orthopaedics, Limb Lengthening and Bone Reconstruction Unit, Rigshospitalet, Copenhagen;; e Department of Orthopaedics, Odense University Hospital, Odense, Denmark

## Abstract

Background and purpose — Observing serious adverse events during treatment with the Precice Stryde bone lengthening nail (NuVasive, San Diego, CA, USA), we conducted a nationwide cross-sectional study to report the prevalence of adverse events from all 30 bone segments in 27 patients treated in Denmark.

Patients and methods — Radiographs of all bone segments were evaluated regarding radiographic changes in February 2021. We determined the number of bone segments with late onset of pain and/or radiographically confirmed osteolysis, periosteal reaction, or cortical hypertrophy in the junctional area of the nail.

Results — In 30 bone segments of 27 patients we observed radiographic changes in 21/30 segments of 20/27 patients, i.e., 19/30 osteolysis, 12/30 periosteal reaction (most often multi-layered), and 12/30 cortical hypertrophy in the area of the junction between the telescoping nail parts. Late onset of pain was a prominent feature in 8 patients. This is likely to be a prodrome to the bony changes. Discoloration (potential corrosion) at the nail interface was observed in multiple removed nails. 15/30 nails were still at risk of developing complications, i.e., were not yet removed.

Interpretation — All Stryde nails should be monitored at regular intervals until removal. Onset of pain at late stages of limb lengthening, i.e., consolidation of the regenerate, should warrant immediate radiographic examination regarding osteolysis, periosteal reaction, and cortical hypertrophy, which may be associated with discoloration (potential corrosion) of the nail. We recommend removal of Stryde implants as early as possible after consolidation of the regenerate.

Bone reconstruction and lengthening surgery entails many risks and unplanned surgeries are common (Frost et al. 2021, Morrison et al. 2020, Sheridan et al. 2020). However, since many adverse events can be managed with or without surgical intervention without affecting the long-term outcome, Paley (1990) redefined complications by subdividing these adverse events into problems, obstacles, and complications. Similarly, other groups suggest grading the severity of adverse events (class I–II–IIIA–IIIB) and dividing these into device and non-device-related complications (Black et al. 2015, Frost et al. 2021). The introduction of an all internal Stryde bone lengthening nail (NuVasive, Specialized Orthopedics, San Diego, CA) in May 2018 was a game changer for bone-lengthening surgery because it enabled the majority of patient to fully weight-bear. Furthermore, the first publications showed promising clinical results with only few device-related complications and good biocompatibility without signs of corrosion (Robbins and Paley 2020, Iliadis et al. 2021).

However, on February 4, 2021 the Danish Medicines Agency released an urgent field safety notice from NuVasive regarding Stryde and all PRECICE system devices. This notice came to prominence based on the British Medicines & Healthcare products Regulatory Agency (MHRA) identifying safety concerns. In the MHRA reference 2020/012/009/226/001 issued January 20, 2021 one concern that was raised was the “unknown long-term biological safety profile. This includes reports of pain and bony abnormalities at the interface between the telescoping nail segments.”

We evaluated the prevalence of radiographic changes in terms of osteolysis, periosteal reactions, and cortical hypertrophy at the junction of the telescoping nail segments as well as late onset of pain and/or swelling in the area.

## Patients and methods

We performed a cross-sectional analysis of all bone-lengthening patients operated on with the Stryde implant at Aarhus University Hospital, Aalborg University Hospital, Odense University Hospital, and Rigshospitalet Denmark.

**Figure 1.**
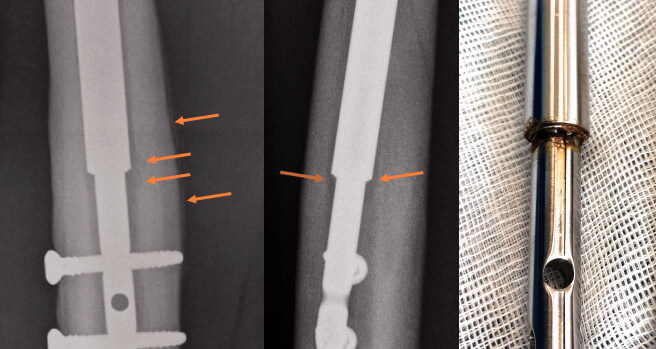
A removed nail. Osteolysis, periosteal reaction, and discoloration (potential corrosion) are evident 315 days after index surgery. Please refer to the Supplementary data for other examples.

3 patients presented with severe adverse events at 3 of the centers on December 15, 2020, January 29, 2021, and February 1, 2021 (Supplementary data). These events and the concomitant field safety notice led to the present study. New radiographs of all implanted nails were obtained in order to assess the prevalence of bony changes on standard anteroposterior and lateral radiographs. The treating physicians reported the clinical findings: pain and/or swelling in the junctional area. The radiographic changes were classified based on a consensus decision of 3 authors evaluating the latest radiographs obtained in January/February 2021 or the latest radiographs before hardware removal (Figure 1, Supplementary data).

Patients were identified by searching the electronic patient records for NOMESCO codes for bone-lengthening surgery of the lower limb (KNFK69 or KNGK69). The number of patients at each center was double-checked with the number of billings from the Danish distributor to either hospital. There were no exclusion criteria.

### Primary outcome measures

- Number of bone segments with confirmed osteolysis and/or periosteal reaction and/or cortical hypertrophy in the junctional area in at least one radiographic projection.
- Late onset of symptoms, i.e., pain and/or swelling in the junctional area with or without radiographic changes.

### Secondary outcome measures

Symptoms warranting unplanned radiographic examination and further clinical and paraclinical investigations; number of patients still at risk, e.g., implant not yet removed; visual inspection and photo documentation of the removed implant.

#### Ethics, funding, and conflicts of interest

The study was conducted according to the Declaration of Helsinki and was approved by the local institutional review board as part of standard clinical care and service evaluation. No external funding was obtained. The authors declare no conflicts of interest.

## Results

30 bone segments (19 femurs, 11 tibias) of 27 patients were lengthened using the fully weight-bearing Stryde nail in Denmark from the release of the Stryde in May 2018 until January 2021. According to the Danish distributor, no other Stryde nails were implanted at other hospitals or private practices in Denmark. We are thus able to present data on all 30 Danish patients. The evaluated radiographs of all 30 nails and clinical photos of removed nails are available as Supplementary data.

Table 1 gives the median values of number of bone segments, number of patients, the age of the patients at the lengthening surgery, the planned bone lengthening, the observation time, and number of nails that have not yet been removed.

**Table 1.** Population characteristics: median (range)

	Total	Aarhus University Hospital	Rigs- hospitalet	Aalborg University Hospital	Odense University Hospital
Bone segments, n	30	12	7	9	2
Femur	19	7	6	4	2
Tibia	11	5	1	5	0
Patients, n	27	9	7	9	2
Patient age	20 (11–65)	18 (11–65)	20 (15–41)	19 (16–42)	17; 46
Planned lengthening, mm	35 (15–80)	40 (15–80)	33 (25–65)	35 (20–80)	20; 35
Evaluated radiograph after					
lengthening procedure, months	11 (2–23)	11 (6–20)	11 (2–20)	8 (3–23)	12; 20
Removed nails, n	15	7	2	5	1
routinely	8	3	1	3	1
due to complication or pain	7	4 **^a^**	1 **^b^**	2 **^c^**	0
Not yet removed nail, n	15	5	5 **^d^**	4	1

**^a^**2 nails—severe pain and osteolysis and periosteal reaction, 1 nail—severe pain only,

1 nail—delayed healing and broken nail.

**^b^**1 nail—pain and osteolysis.

**^c^**1 nail—severe pain and osteolysis and periosteal reaction,

1 nail—valgus deformity corrected with trauma nail.

**^d^**1 nail—delayed healing and broken nail, not yet removed.

### Primary outcome measures

**Figure 2.**
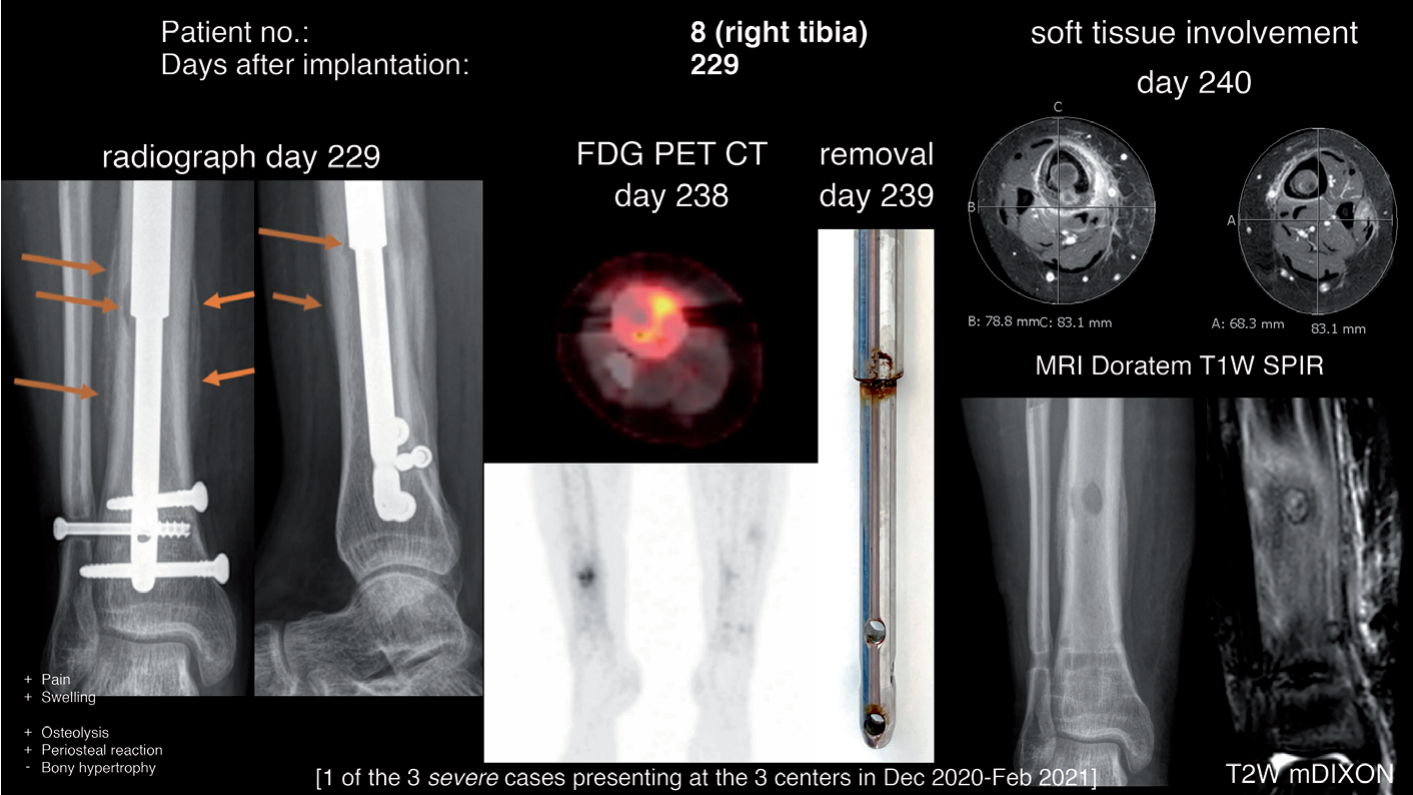
Osteolysis and periosteal reaction at postoperative day 229 (48 days after the onset of symptoms, i.e., pain in the area). FDG PET CT revealed increased glucose uptake in the area. The removed nail (day 239) had discoloration (potential corrosion) and on MRI on the subsequent day cortical destruction and periosteal reaction of the right tibia as well as soft tissue involvement were present. In comparison, a Stryde nail without discoloration was simultaneously removed from the asymptomatic left side.

Evaluating the latest radiographs of 30 nails, we found 19/30 osteolysis, 12/30 periosteal reaction, 12/30 cortical hypertrophy (Supplementary data). 9/30 lengthened bone segments had no radiographic junctional changes, while 1 radiographic abnormality was present in 4/30 and 17/30 had 2 or all 3 radiographic signs. Periosteal reactions were most often of multilayered onion-skin type, indicative of rapid evolvement, and may thus resemble tumor or infection (Figure 2).

Late onset of pain in the area was prevalent in 6 of these patients with verified radiographic changes. Furthermore, 2 patients complained of late onset of pain without any radiographic signs present.

Figure 1 illustrates osteolysis and periosteal reaction after consolidation of the regenerate as well as discoloration (potential corrosion) after removal of a nail. This observation was present in many of the removed nails (Supplementary data).

Figure 2 highlights the time-dependency of the observed adverse event with neither sign of osteolysis nor periosteal reaction 11 weeks before the fulminant appearance. Figure 2 also demonstrates that the radiographic signs can mimic infection or tumor. Cortical destruction and marked periosteal reaction, as well as soft-tissue involvement and swelling, are evident on the postoperative MRI findings of the right compared with the asymptomatic left tibia of the same patient, who had both Stryde nails (right side with discoloration, left side without discoloration) removed simultaneously. Importantly, none of the obtained biopsies were culture-positive. However, a biopsy from the medullary cavity revealed an inflammatory reaction (acute/chronic) with foreign-body giant cells and metallic material. Similar findings were made in patient no. 17 (Supplementary data).

Unrelated to the scope of the study, but importantly, 2/30 femoral Stryde nails broke within 1 year after implantation (nail diameter: Ø11.5, Ø11.5; patient weight: 55 kg, 85 kg).

## Discussion

In this cross-sectional study, the prevalence of either osteolysis, periosteal reaction, or cortical hypertrophy was 21/30 in the area of the telescoping interface of the Stryde limb lengthening nail. If the suspected prodrome—late onset of pain in the junctional area—is included, 23/30 of segments were affected. Despite these alarming numbers, the true incidence rate is likely to be higher, as 15/30 of the bone segments were still at risk, i.e., the nails were not removed at the time of writing.

The 1st case with minor osteolytic lesions occurred in May 2020 and the radiographic changes were retrospectively identified following the presentation of the more pronounced cases and the Field Safety Notice from NuVasive Specialized Orthopedics in Denmark from February 4, 2021 (Danish Medicines Agency 2021). The 3 cases with more pronounced bony reactions presented at 3 different centers within 7 weeks from the middle of December 2020. Our findings are in line with the MHRA statement: “reports of pain and bony abnormalities at the interface between the telescoping nail segments”. In contrast, Robbins and Paley (2020), who were the first to implant the device in May 2018, state in their paper from 2020 evaluating 187 lengthened bone segments in 106 patients: “There were no issues related to biological incompatibility of the Biodur® 108 alloy stainless steel from which the implant was fabricated. There was no corrosion seen in the few nails that were removed during this short study time.”

Biodur® 108 Alloy (ASTM F2229) is an alloy almost free from nickel and carbon, which theoretically should be stronger and more corrosion resistant compared with stainless steel and other alloys, thus allowing for full weight-bearing during the lengthening process (Robbins and Paley 2020). According to ASTM F2229 the composition of the alloy (% mass/mass) is manganese 21–24, chromium 19–23, molybdenum 0.5–1.5, nitrogen 0.85–1.1, silicon 0.75 max, carbon 0.08 max, nickel 0.05 max, phosphorus 0.03 max, copper 0.25 max, sulfur 0.01 max, and iron.

**Figure 3.**
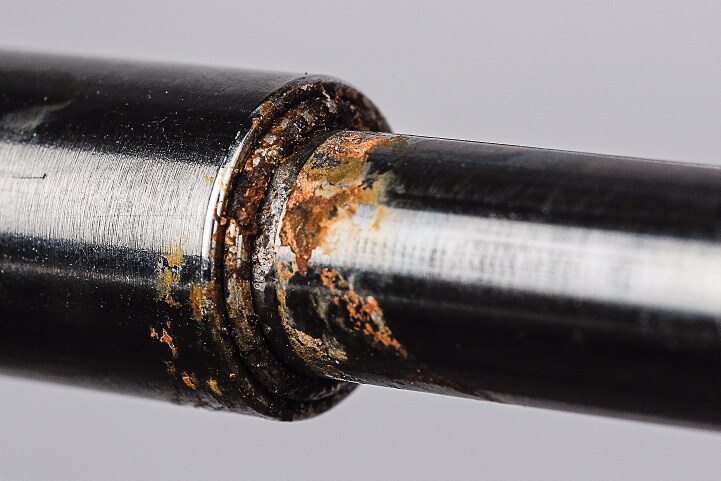
Discoloration at the telescoping interface of a removed Stryde nail.

We observed discoloration (potential corrosion) at the junction of the telescoping parts of the symptomatic patients (Figure 3, Supplementary data). Despite these observations the reason for the reported symptoms and radiographic changes remains speculative. Importantly, no nails were recharged or backed, which potentially could have worn a seal, etc. (Panagiotopoulou et al. 2018, Schiedel 2020).

The adverse events predominantly occurred during late stages of bone lengthening, i.e., after (or during) maturation of the regenerate and after the structural integrity of the bone has been restored (Hvid et al. 2016). Micromotion and wear debris at the nail junction should therefore have been minimal when the adverse events were noticed. Whether full weight-bearing during earlier stages may have set processes in motion causing the later onset of symptoms, or whether the alloy is not protecting against, but rather causing corrosion has yet to be determined. However, other explanations may also apply. The affected nails were or will be further analyzed, which may offer an explanation at a later stage.

The marked response of the bone tissue with intramedullary osteolysis and periosteal reaction was in 6 cases preceded by pain, and 2 patient were suddenly unable to fully weight-bear after solid consolidation of the regenerate. Whether the observed changes in the majority of cases are confined to the bony tissue or if the surrounding soft tissue also is affected as demonstrated in Figure 2 is unclear. Soft tissue biopsies and/or MRI scans after removal of the nail may shed light on this.

The time from onset of symptoms to radiographic changes ranged from 15 to 48 days. In particular, the manifestation of fulminant radiologic changes within 11 weeks between normal appearing bone to pronounced intra- and extramedullary bony changes is concerning (Figure 2). Pain and later swelling had been present for approximately 7 weeks prior to the radiographic changes. However, despite these symptoms the patient did not contact the treating physician before the scheduled clinical and radiographic control in the outpatient clinic. Notably, radiographic changes were also seen in patients with no or subclinical symptoms.

Vogt et al. (2021) also describe osteolysis in Stryde nails. Late onset of pain was also the dominant clinical feature in their case series, which was relieved by hardware removal. Moreover, they observed discoloration of the nail at the telescoping interface.

All our patients were within the weight-bearing limit of the applied nail (Robbins and Paley 2020). Nonetheless, 2 femoral Stryde nails broke within 1 year after implantation and causing reoperations in this series of 30 nails. Both patients were well within the weight limit of the Ø11.5 mm femoral nail, i.e., 55 kg and 85 kg. This adverse event is not considered to be related to the described osteolysis, but a consequence of regenerate insufficiency.

Unrelated to these adverse events, the MHRA also raised concerns about a potentially “inappropriate use in children and adolescents” (British Orthopaedic Association 2021). The MHRA acknowledges a widespread use of PRECICE devices in this age group, but highlights that the nails have not been validated for use in these patient groups (Frommer et al. 2018, Nasto et al. 2020, Iobst 2020, Iliadis et al. 2021, Vogt et al. 2021). In our study, 10/27 patients were younger than 18 years old with the youngest patient being 11 years of age.

Unlike the Stryde, other bone-lengthening nails such as the titanium PRECICE lengthening nail (introduced in May 2013 and improved to its latest version P2.2. in 2015) and the stainless-steel lengthening nail, Fitbone (Orthofix, Lewisville, TX, USA) have been on the market for many years. The growing body of literature regarding complications of all internal limb lengthening does not state any similar adverse events (Calder et al. 2019, Frost et al. 2021, Morrison et al. 2020, Thaller et al. 2020, Iliadias et al. 2021). However, the current MHRA recommendations not to implant the PRECICE P2.2 as well as scientific scrutiny demand retrospective studies evaluating whether this phenomenon/adverse event also exists on a lower scale with these products. Moreover, the PRECICE Bone Transport nail (Kähler Olesen and Herzenberg 2020, Ferner et al. 2020, Abood et al. 2021) and the bone transport plate are made of the same Biodur 108 alloy, which could be one possible reason for the described phenomenon. Thus, these implants are more likely to be affected than the titanium PRECICE P2.2 (Calder et al. 2019, Kähler Olesen et al. 2019, Nasto et al. 2020) or stainless-steel Fitbone (Krieg et al. 2011, Accadbled et al. 2016, Horn et al. 2019).

### Our surveillance and pre-emptive treatment strategy of the Stryde implant

1. We recommend monitoring patients with Stryde implants closely (4–6 weekly) even, or especially, during late stages of the bone-lengthening process.
2. Patients should be informed about the adverse events and that we consider pain and potential swelling at the junctional area to be prodromes.
3. Hardware removal after the regenerate is sufficiently consolidated should not be delayed, even in asymptomatic patients without radiological changes. Exchange nailing before consolidation may be considered, if exchange nailing was part of the initial treatment plan or in cases with delayed healing or in symptomatic patients.
4. Intramedullary or transcortical bone and soft tissue biopsies may help to identify the cause and extent of this complication.
5. PET-CT may be useful in diagnosis and MRI after hardware removal may also be used in addition to biopsies to rule out resembling diagnosis, i.e., infection and cancer (Figure 2).
6. Blood/urine samples for metals were collected only sporadically in our patients. Similar to the lessons learned from metal-on-metal prostheses, a more structured surveillance program and a consensus on this program should be obtained. However, bone-lengthening nails are extra-articular devices and unlike metal-on-metal prostheses should be removed after bony consolidation. Thus, the risk of the described phenomenon and its potential long-term consequences, e.g., locally within the bone and soft tissue (potentially including pathological fractures) and systemically (metal allergy, deposition of metals in internal organs) may be alleviated by nail removal (Jakobsen et al. 2007, Langton et al. 2010, Hjorth et al. 2016).

In conclusion, osteolysis, periosteal reactions and/or cortical hypertrophy at the telescoping interface were present in 21/30 of the implanted Stryde nails. If the suspected prodrome—late onset of pain and/or swelling in the area—is included, 23/30 segments were affected. Despite these alarming numbers, the true incidence rate is likely to be higher, as 15/30 of the bone segments were still at risk, i.e., the nail was not yet removed. The company has recalled all Stryde implants; however, structured clinical surveillance programs of patients with implanted Stryde nails are warranted.

## Supplementary Material

Supplemental Material

## Supplementary data

Supplementary data is available in the online version of this article. The pdf file includes all 30 evaluated AP and lateral radiographs from January/February 2021 or the latest before nail removal. The consensus decision of 3 authors regarding the presence (+/-) of osteolysis, periosteal reaction, or cortical hypertrophy is given for each evaluated radiograph. Additionally, pathological evaluations of two biopsies are available, see online version of this article, http://dx.doi.org/10.1080/17453674.2021.1903278

Abood A A-H, Petruskevicius J, Vogt B, Frommer A, Rödl R, Rölfing J D. The Joint Angle Tool for intraoperative assessment of coronal alignment of the lower limb. Strategies Tauma Limb Reconstr 2021; epub ahead of print. doi: 10.5005/jp-journals-10080-1511
