# Supplementary material for: Pain, osteolysis, and periosteal reaction are associated with the STRYDE limb lengthening nail: a nationwide cross-sectional study
Source: Acta Orthop. 2021 Mar 24;92(4):479–84. doi: 10.1080/17453674.2021.1903278 (PMC8428270; doi:10.1080/17453674.2021.1903278)
Supplement: Supplemental Material [file IORT_A_1903278_SM7852.pdf]

Supplementary data

This supplementary material consists of radiographs and consensus decisions of 30 segments in 27 patients.

The evaluated radiographs are in ascending order (number of days after implantation). Patients no. 4, 8, 17 were the severe cases presenting with pain and identified radiographic changes at 3 different centers within 7 weeks. All other radiographic changes at the telescoping interfaces were identified retrospectively, including patients 15 and 24 who presented with severe pain, which was relieved by hardware removal.

Bone segment	Patient number	Evaluated radiographs [days]	CLINICAL FEATURES			RADIOGRAPHIC CHANGES			
			Pain	Swelling	*In total*	Osteolysis	Periosteal reaction	Hypertrophy	*In total*
			8/30 (27%)	3/30 (10%)		19/30 (63%)	12/30 (40%)	12/30 (40%)	
1	1	63	0	0	0	0	0	0	0
2	2	81	0	0	0	0	1	0	**1**
3	3	94	0	0	0	0	0	0	0
4	4	136	1	1	2	1	1	0	**2**
5	5	141	0	0	0	1	1	0	**2**
6	6	181	1	0	1	1	1	1	**3**
7	7	187	0	0	0	0	0	0	0
8	8 right tibia	229	1	1	2	1	1	0	**2**
9	8 left tibia	250	0	0	0	0	0	0	0
10	9	231	0	0	0	1	0	1	**2**
11	10	253	0	0	0	0	0	0	0
12	11	273	0	0	0	1	1	1	**3**
13	12	275	0	0	0	1	1	0	**2**
14	13	276	0	0	0	0	1	0	**1**
15	14	299	0	0	0	1	1	0	**2**
16	15	315	1	0	1	1	1	1	**3**
17	16	324	0	0	0	0	0	0	0
18	17	339	1	0	1	1	0	1	**2**
19	18	348	1	0	1	0	0	0	**0**
20	19	360	0	0	0	1	0	1	**2**
21	20	363	1	0	1	1	0	1	**2**
22	21	371	0	0	0	0	0	0	0
23	22	375	0	0	0	1	0	1	**2**
24	23	395	0	0	0	1	0	1	**2**
25	24 right femur	400	0	0	0	1	1	1	**3**
26	24 left femur	491	0	1	1	1	1	1	**3**
27	24 right tibia	593	1	0	1	0	0	0	0
28	25	545	0	0	0	1	0	0	**1**
29	26	609	0	0	0	1	0	0	**1**
30	27	610	0	0	0	1	0	1	**2**

Bone segments with pain and swelling

neither	21 /30 (70%)
either / or	7 /30 (23%)
both	2 /30 (. 7%)

Number of radiographic changes per segment

0	9 /30 (30%)
1	4 /30 (13%)
2	12 /30 (40%)
3	5 /30 (17%)

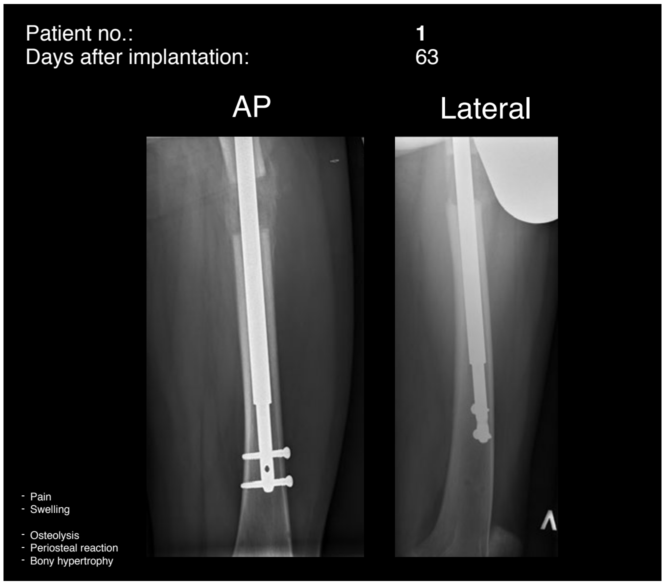
Patient no.: 1
Days after implantation: 63
AP
Lateral
- Pain
- Swelling
- Osteolysis
- Periosteal reaction
- Bony hypertrophy

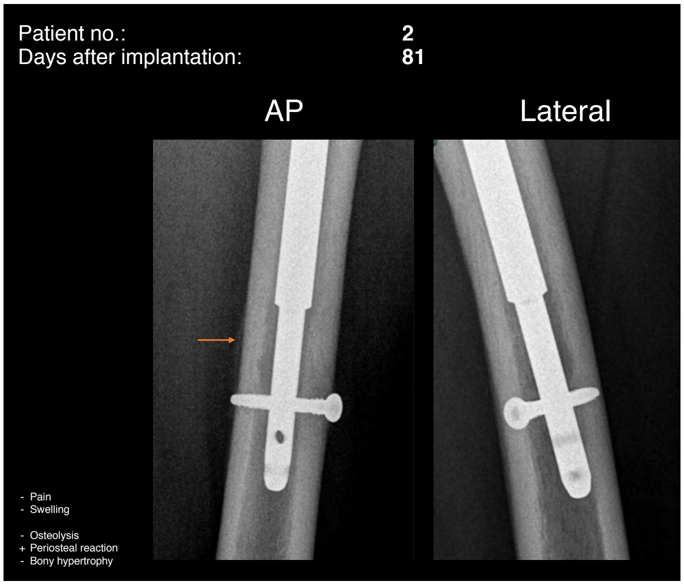
Patient no.: 2
Days after implantation: 81
AP
Lateral
- Pain
- Swelling
- Osteolysis
+ Periosteal reaction
- Bony hypertrophy

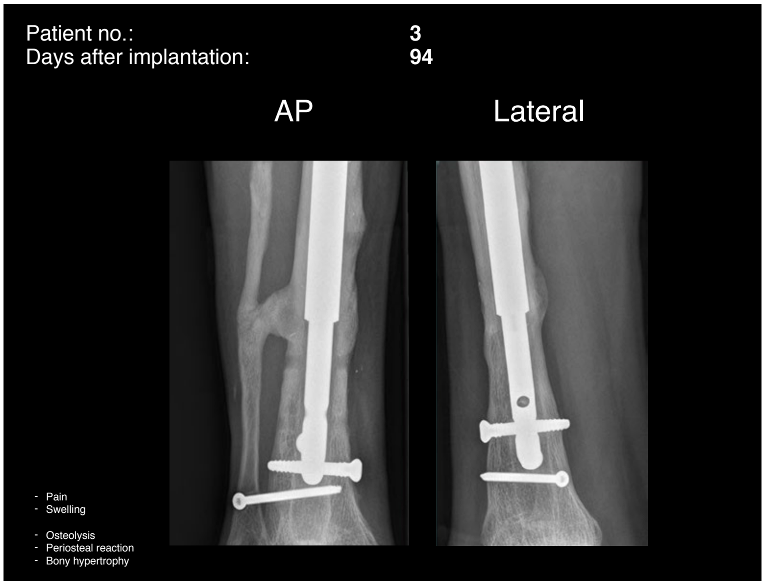
Patient no.: 3
Days after implantation: 94
AP
Lateral
- Pain
- Swelling
- Osteolysis
- Periosteal reaction
- Bony hypertrophy

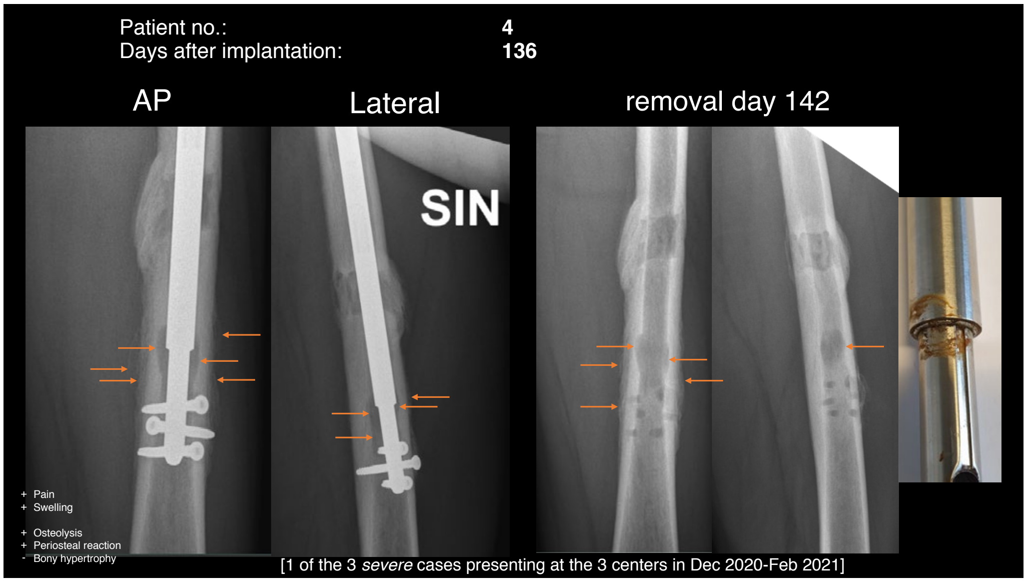
Patient no.: 4
Days after implantation: 136
AP
Lateral
removal day 142
SIN
+ Pain
+ Swelling
+ Osteolysis
+ Periosteal reaction
- Bony hypertrophy
[1 of the 3 *severe* cases presenting at the 3 centers in Dec 2020-Feb 2021]

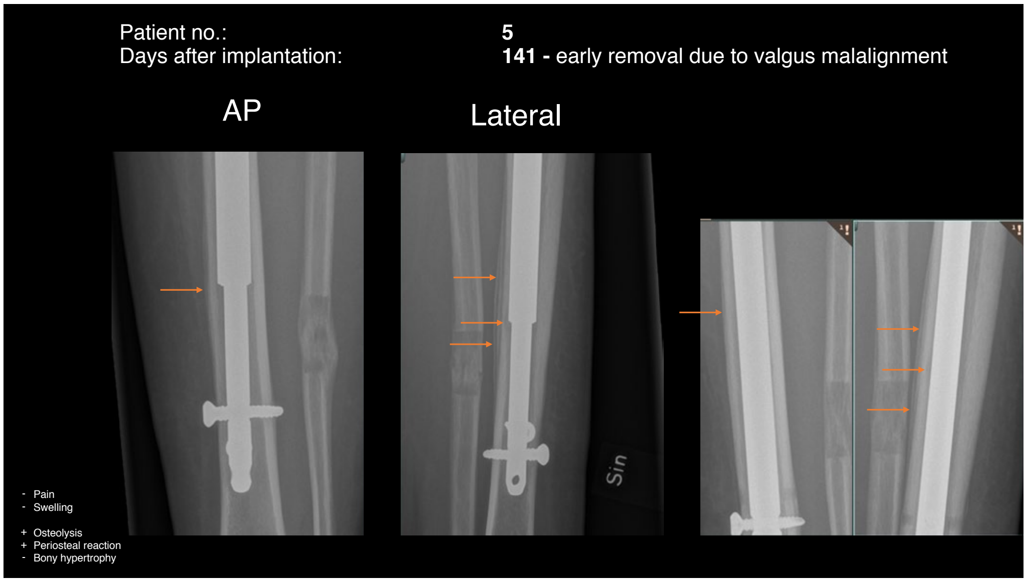
Patient no.: 5
Days after implantation: 141 - early removal due to valgus malalignment
AP
Lateral
- Pain
- Swelling
+ Osteolysis
+ Periosteal reaction
- Bony hypertrophy

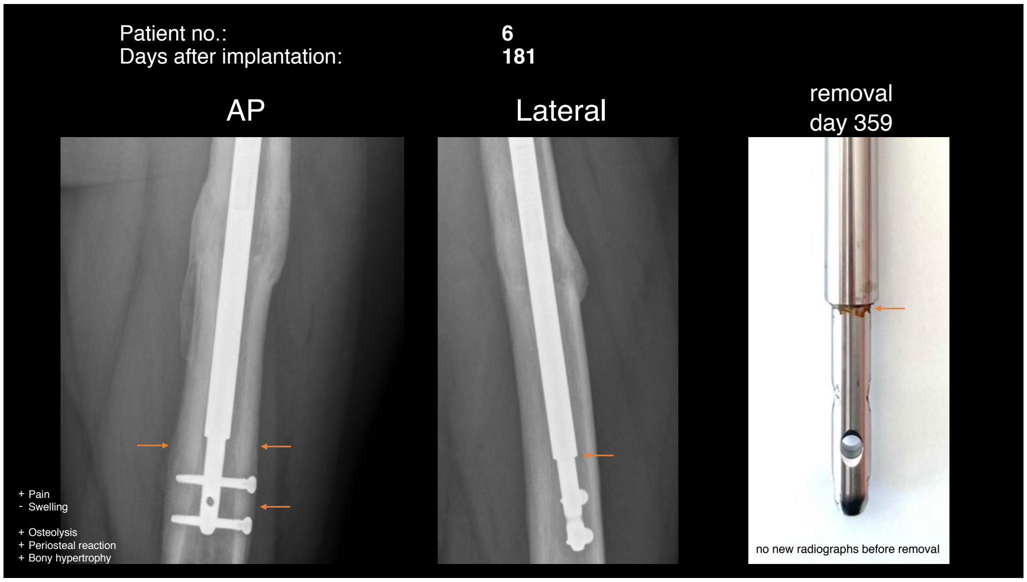
Patient no.: 6
Days after implantation: 181
AP
Lateral
removal
day 359
no new radiographs before removal
+ Pain
- Swelling
+ Osteolysis
+ Periosteal reaction
+ Bony hypertrophy

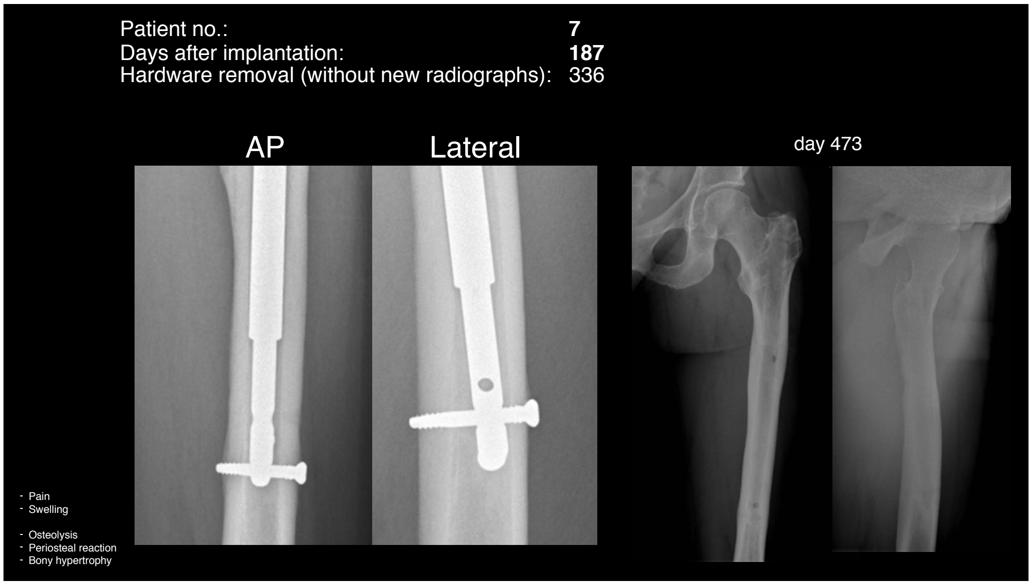
Patient no.: 7
Days after implantation: 187
Hardware removal (without new radiographs): 336
AP
Lateral
day 473
- Pain
- Swelling
- Osteolysis
- Periosteal reaction
- Bony hypertrophy

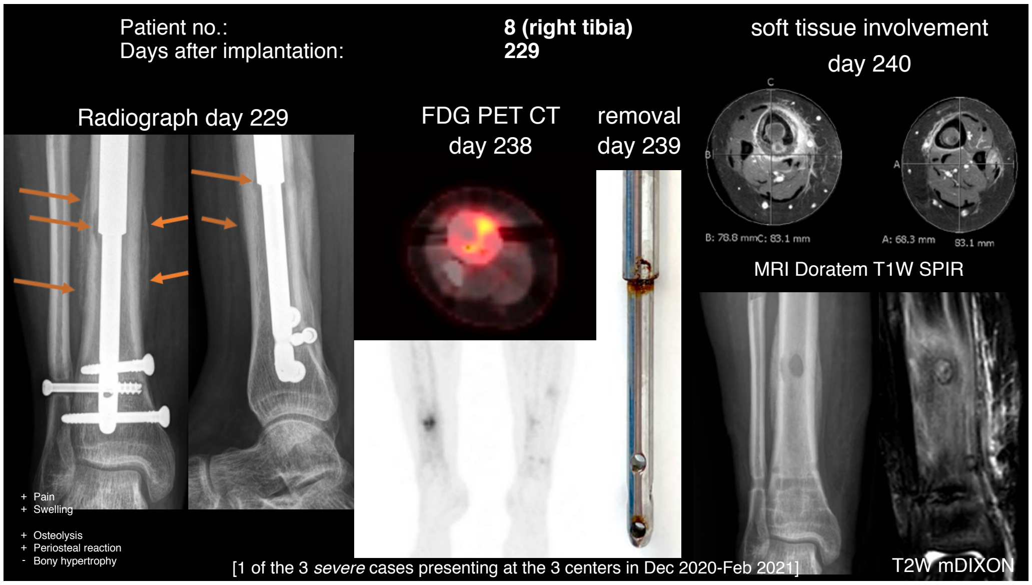
Patient no.:
Days after implantation:
8 (right tibia)
229
soft tissue involvement
day 240
Radiograph day 229
FDG PET CT
day 238
removal
day 239
MRI Doratem T1W SPIR
+ Pain
+ Swelling
+ Osteolysis
+ Periosteal reaction
- Bony hypertrophy
[1 of the 3 severe cases presenting at the 3 centers in Dec 2020-Feb 2021]
T2W mDIXON

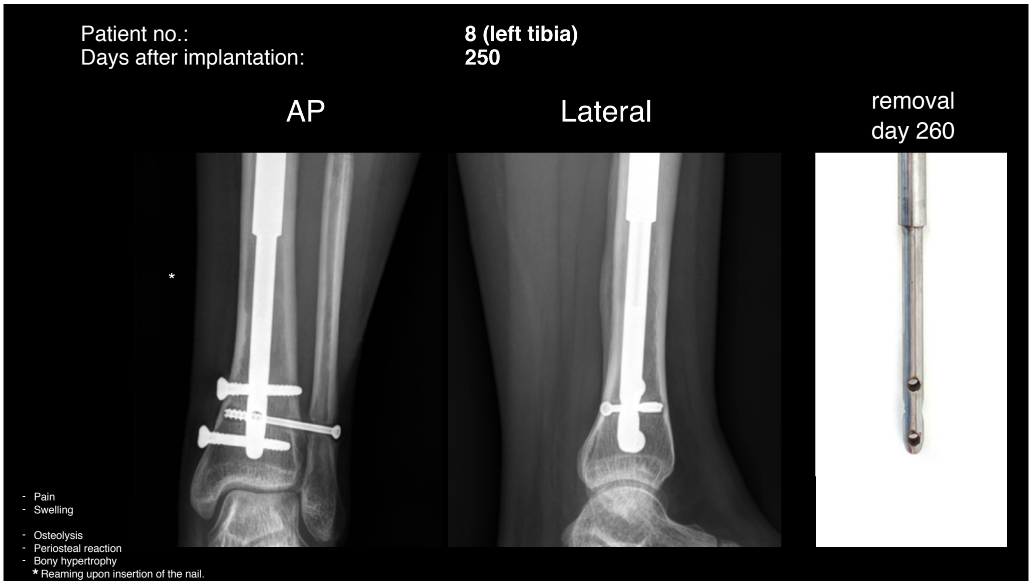
Patient no.:
Days after implantation:
8 (left tibia)
250
AP
Lateral
removal
day 260
*
- Pain
- Swelling
- Osteolysis
- Periosteal reaction
- Bony hypertrophy
* Reaming upon insertion of the nail.

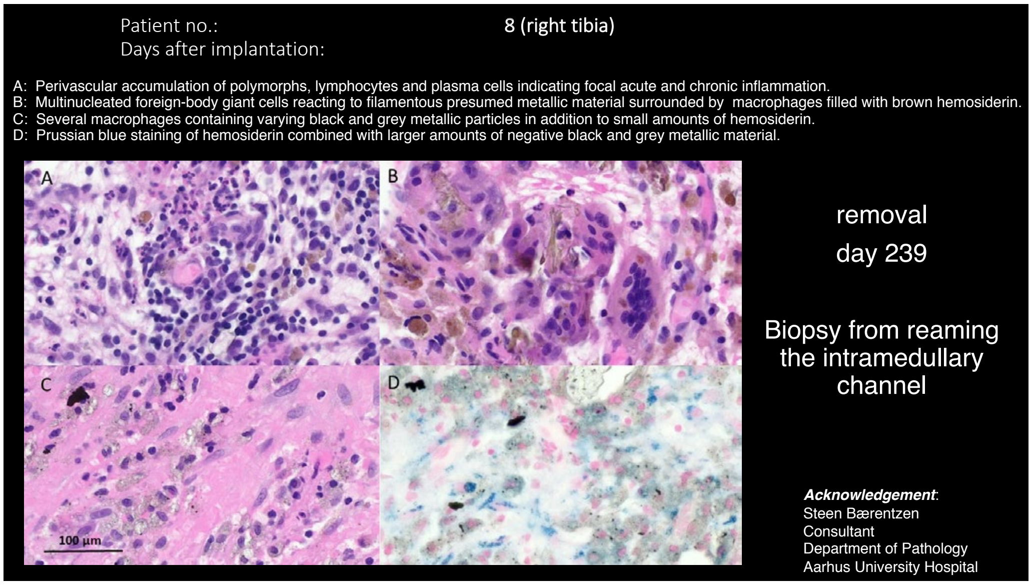
Patient no.:
Days after implantation:
8 (right tibia)
A: Perivascular accumulation of polymorphs, lymphocytes and plasma cells indicating focal acute and chronic inflammation.
B: Multinucleated foreign-body giant cells reacting to filamentous presumed metallic material surrounded by macrophages filled with brown hemosiderin.
C: Several macrophages containing varying black and grey metallic particles in addition to small amounts of hemosiderin.
D: Prussian blue staining of hemosiderin combined with larger amounts of negative black and grey metallic material.
A
B
C
D
100 µm
removal
day 239
Biopsy from reaming the intramedullary channel
Acknowledgement:
Steen Bærentzen
Consultant
Department of Pathology
Aarhus University Hospital

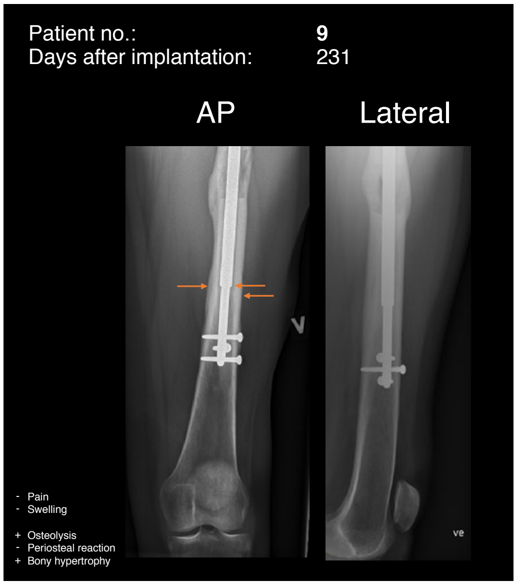
Patient no.: 9
Days after implantation: 231
AP
Lateral
- Pain
- Swelling
+ Osteolysis
- Periosteal reaction
+ Bony hypertrophy

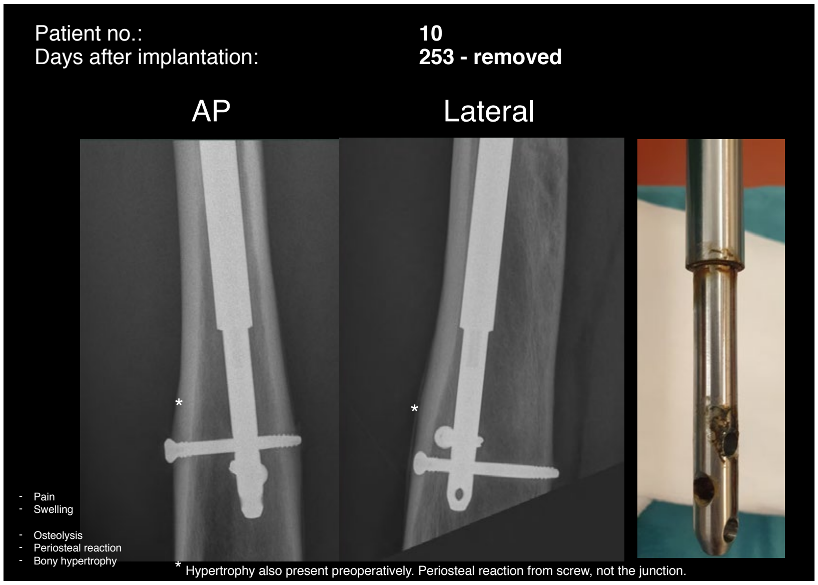
Patient no.: 10
Days after implantation: 253 - removed
AP
Lateral
- Pain
- Swelling
- Osteolysis
- Periosteal reaction
- Bony hypertrophy
* Hypertrophy also present preoperatively. Periosteal reaction from screw, not the junction.

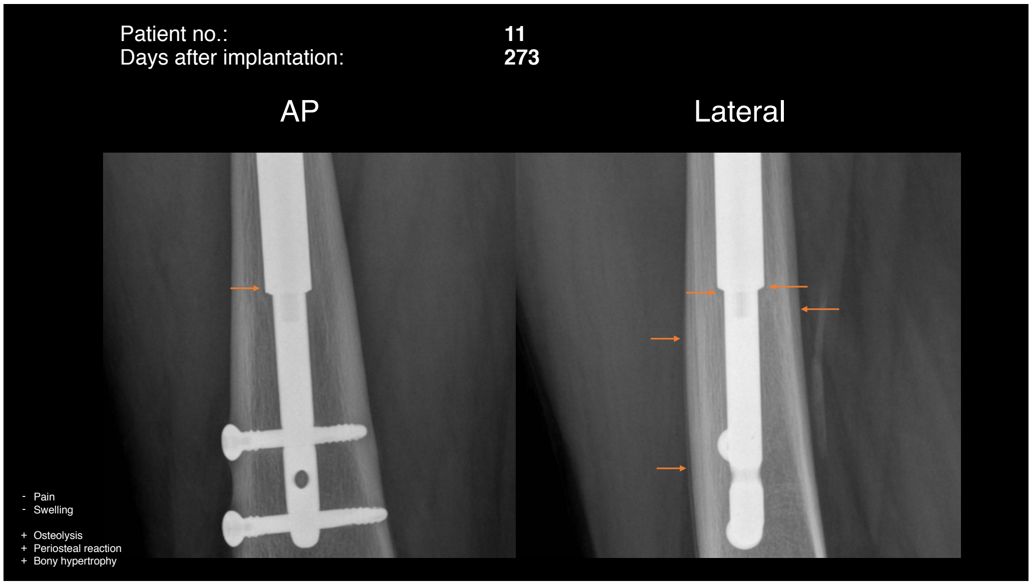
Patient no.: 11
Days after implantation: 273
AP
Lateral
- Pain
- Swelling
+ Osteolysis
+ Periosteal reaction
+ Bony hypertrophy

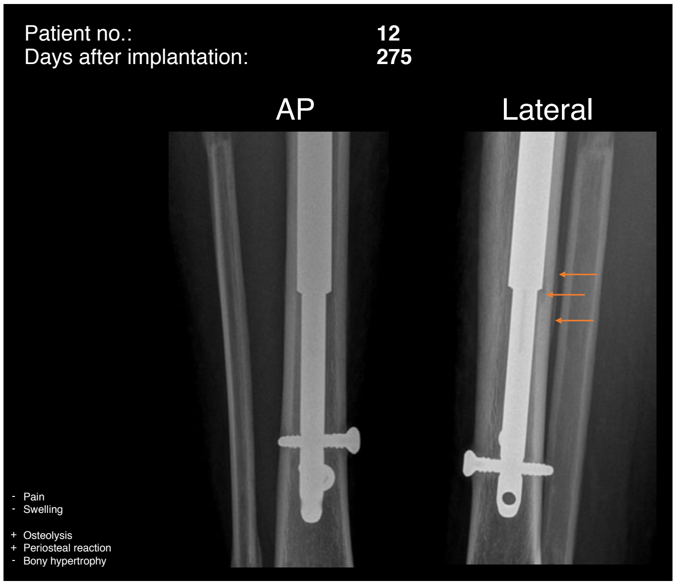
Patient no.: 12
Days after implantation: 275
AP
Lateral
- Pain
- Swelling
+ Osteolysis
+ Periosteal reaction
- Bony hypertrophy

Supplementary data (6/11) *Acta Orthopaedica* 2021; 92 (DOI: 10.1080/17453674.2021.1903278)

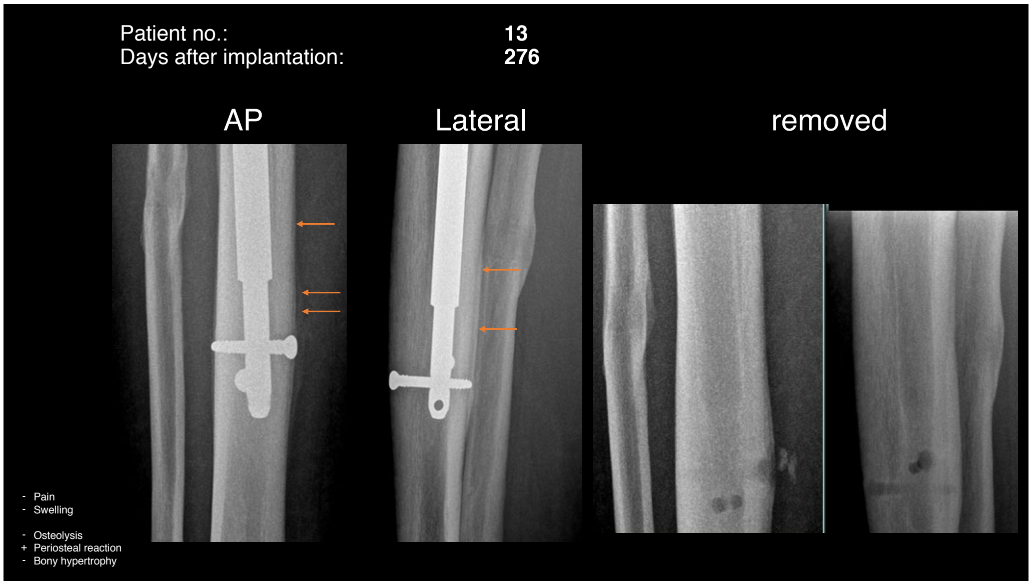

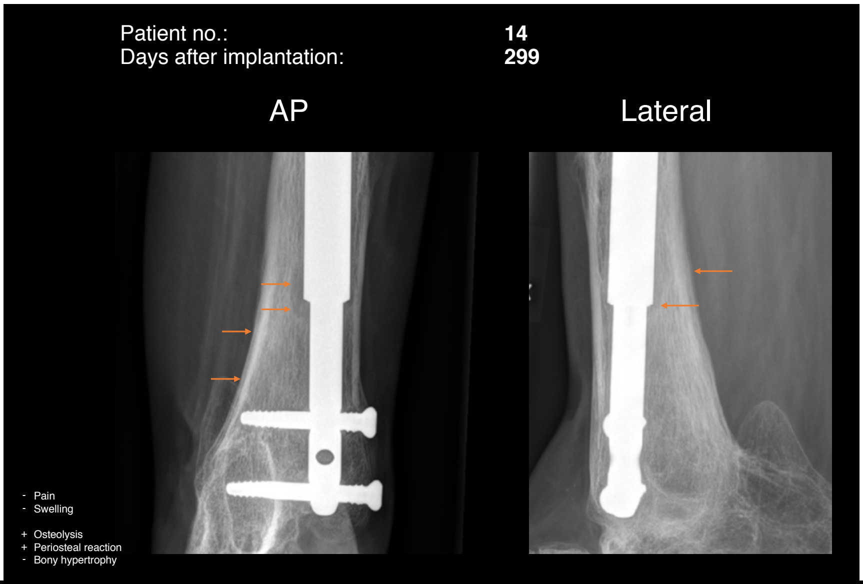

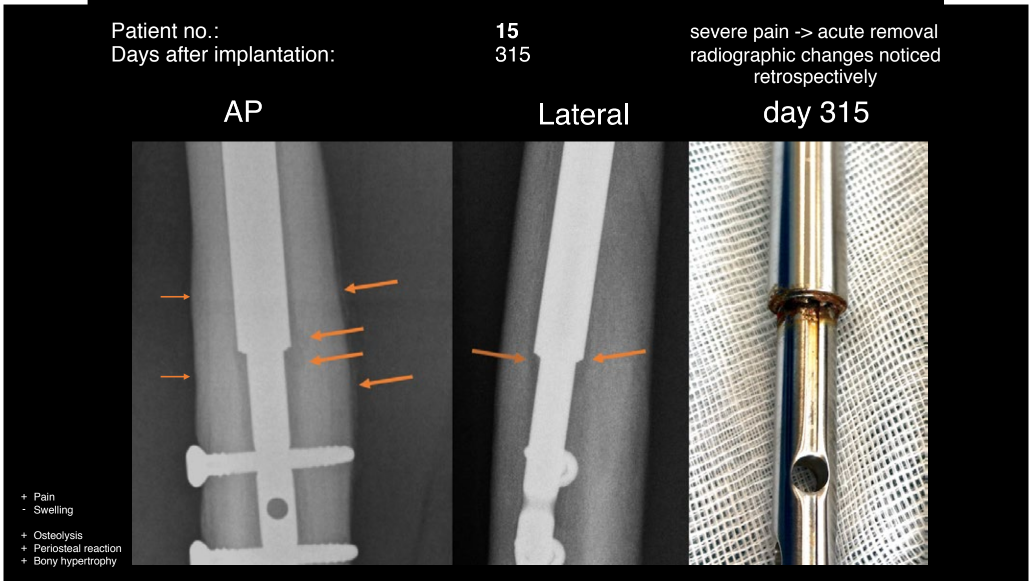

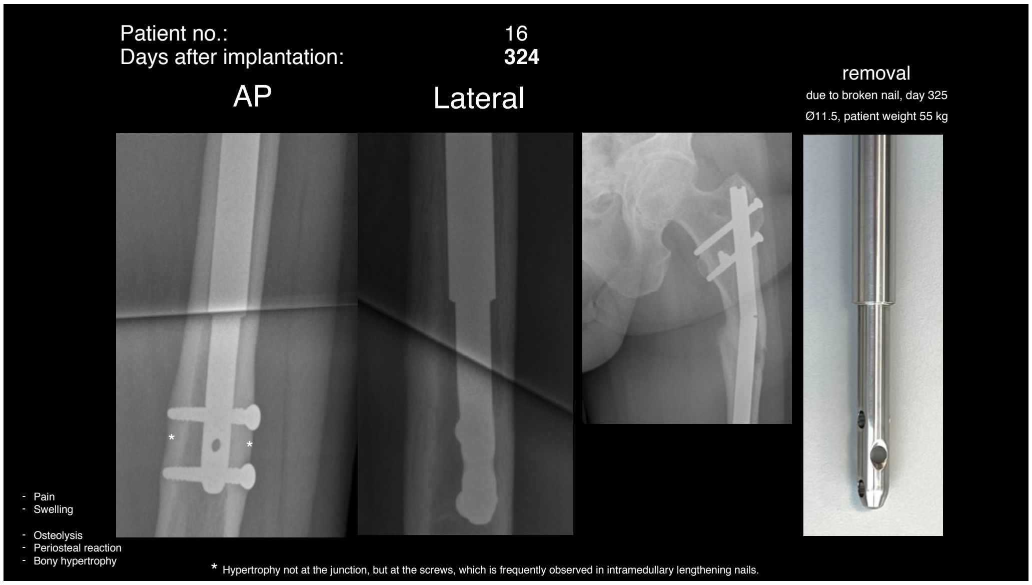
Patient no.: 16
Days after implantation: 324
AP
Lateral
removal
due to broken nail, day 325
Ø11.5, patient weight 55 kg
- Pain
- Swelling
- Osteolysis
- Periosteal reaction
- Bony hypertrophy
* Hypertrophy not at the junction, but at the screws, which is frequently observed in intramedullary lengthening nails.

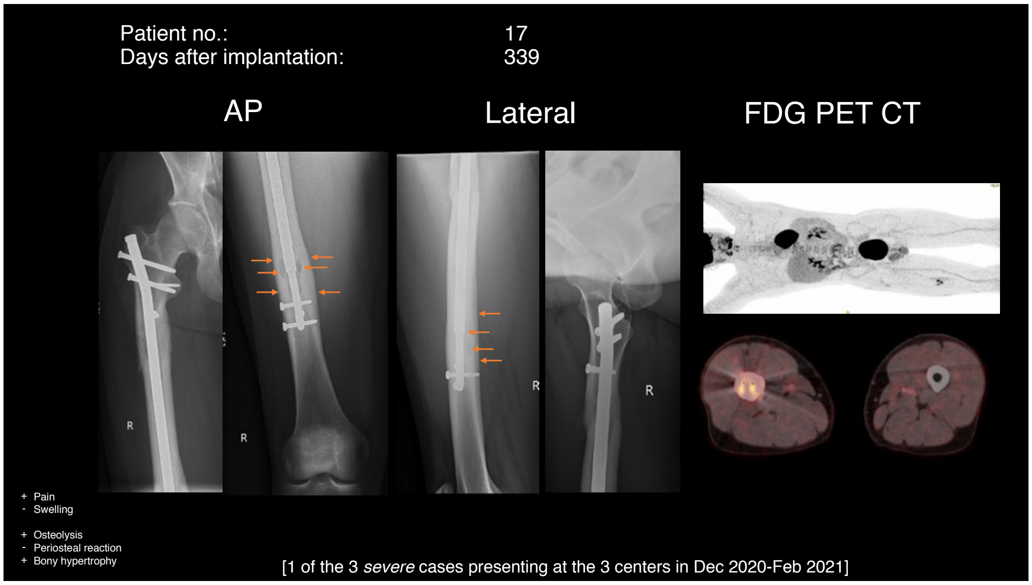
Patient no.: 17
Days after implantation: 339
AP
Lateral
FDG PET CT
+ Pain
- Swelling
+ Osteolysis
- Periosteal reaction
+ Bony hypertrophy
[1 of the 3 *severe* cases presenting at the 3 centers in Dec 2020-Feb 2021]

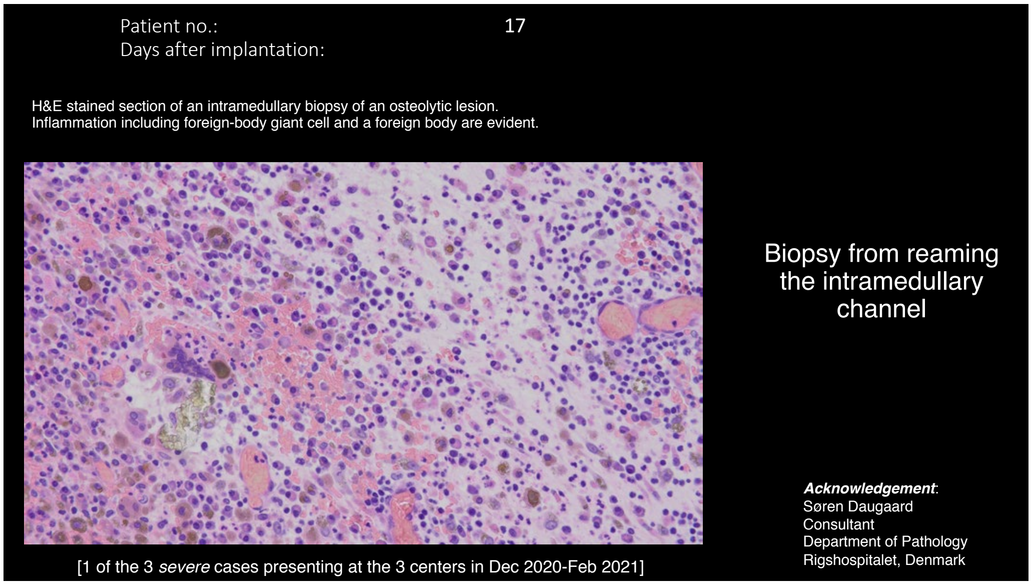
Patient no.: 17
Days after implantation:
H&E stained section of an intramedullary biopsy of an osteolytic lesion.
Inflammation including foreign-body giant cell and a foreign body are evident.
Biopsy from reaming the intramedullary channel
[1 of the 3 *severe* cases presenting at the 3 centers in Dec 2020-Feb 2021]
Acknowledgement:
Søren Daugaard
Consultant
Department of Pathology
Rigshospitalet, Denmark

Patient no.: **18**
Days after implantation: 348

AP Lateral

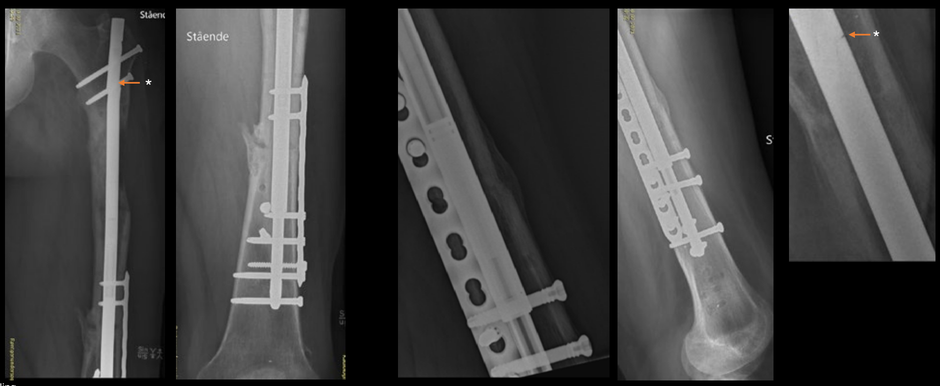

+ Pain
- Swelling

- Osteolysis
- Periosteal reaction
- Bony hypertrophy

* broken nail Ø11.5, patient weight: 85 kg (not yet removed), delayed healing

Patient no.: **19**
Days after implantation: 360 (+ 90 after removal)

AP Lateral

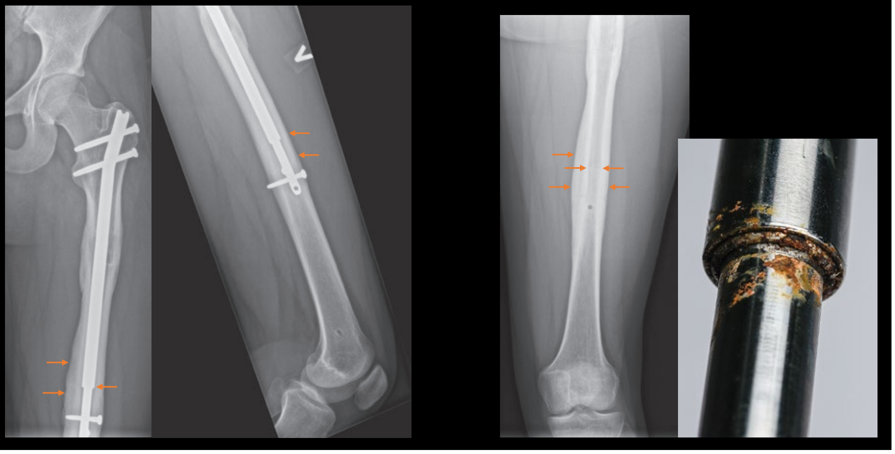

- Pain
- Swelling

+ Osteolysis
- Periosteal reaction
+ Bony hypertrophy

Patient no.: **20**
Days after implantation: **363**

AP Lateral

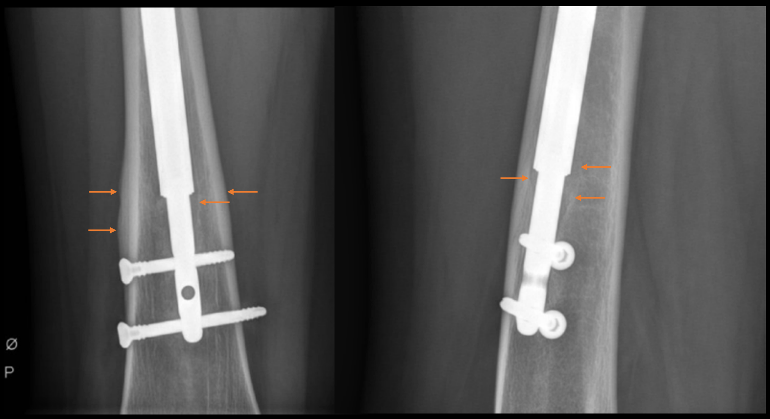

+ Pain
- Swelling

+ Osteolysis
- Periosteal reaction
+ Bony hypertrophy

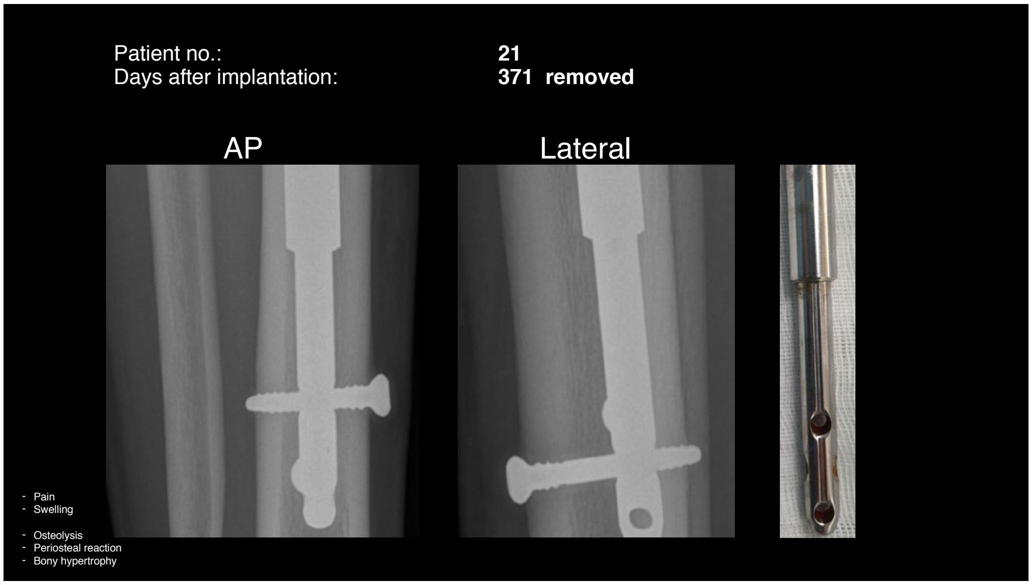
Patient no.: **21**
Days after implantation: **371 removed**
AP
Lateral
- Pain
- Swelling
- Osteolysis
- Periosteal reaction
- Bony hypertrophy

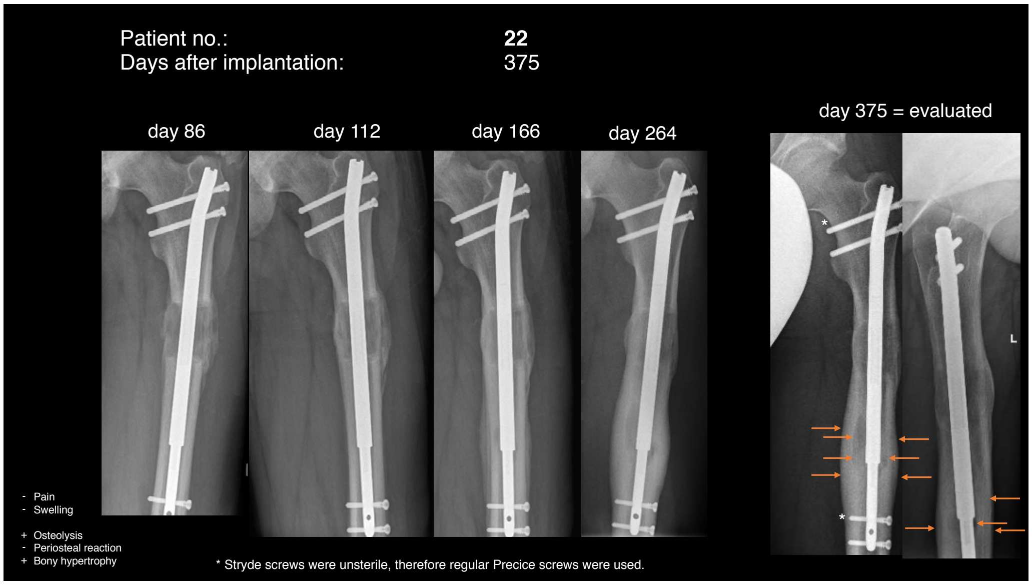
Patient no.: **22**
Days after implantation: 375
day 86
day 112
day 166
day 264
day 375 = evaluated
L
- Pain
- Swelling
+ Osteolysis
- Periosteal reaction
+ Bony hypertrophy
* Stryde screws were unsterile, therefore regular Precice screws were used.

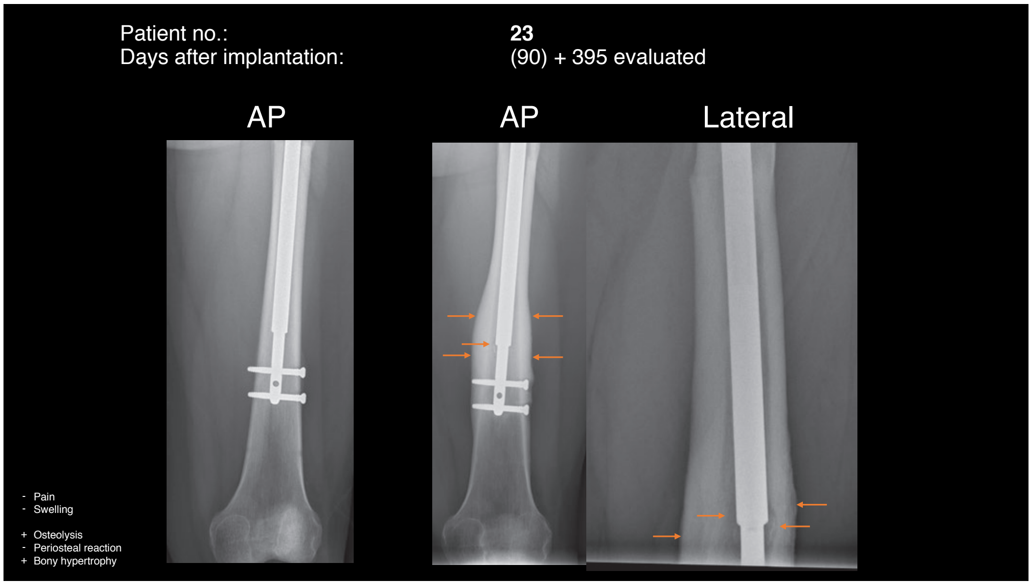
Patient no.: **23**
Days after implantation: (90) + 395 evaluated
AP
AP
Lateral
- Pain
- Swelling
+ Osteolysis
- Periosteal reaction
+ Bony hypertrophy

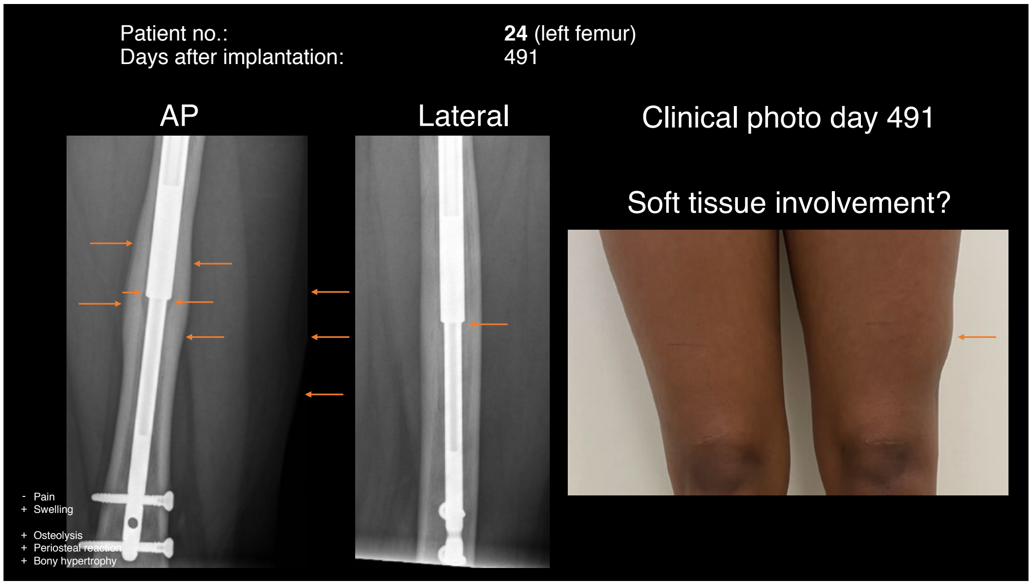
Patient no.: 24 (left femur)
Days after implantation: 491
AP
Lateral
Clinical photo day 491
Soft tissue involvement?
- Pain
+ Swelling
+ Osteolysis
+ Periosteal reaction
+ Bony hypertrophy

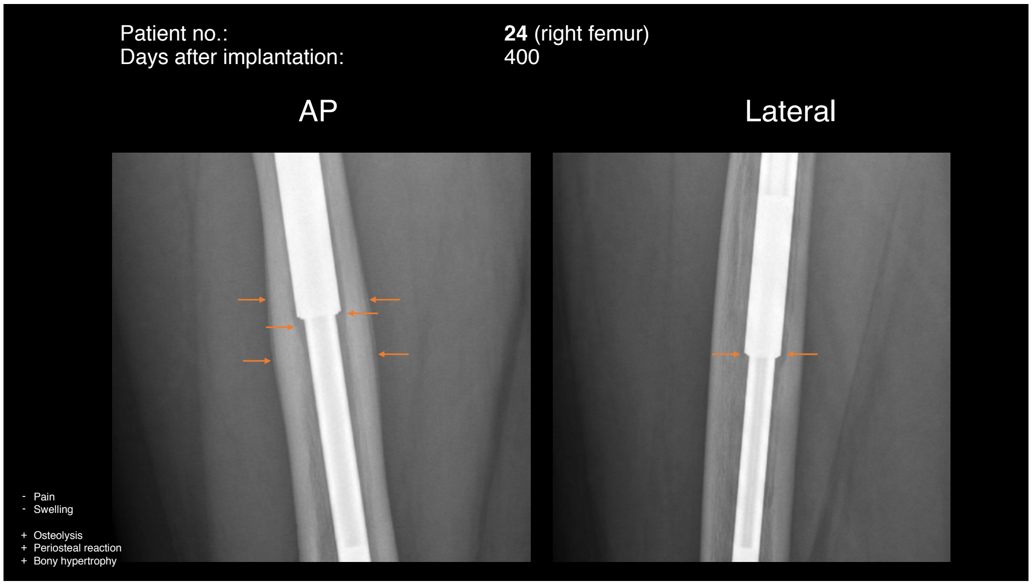
Patient no.: 24 (right femur)
Days after implantation: 400
AP
Lateral
- Pain
- Swelling
+ Osteolysis
+ Periosteal reaction
+ Bony hypertrophy

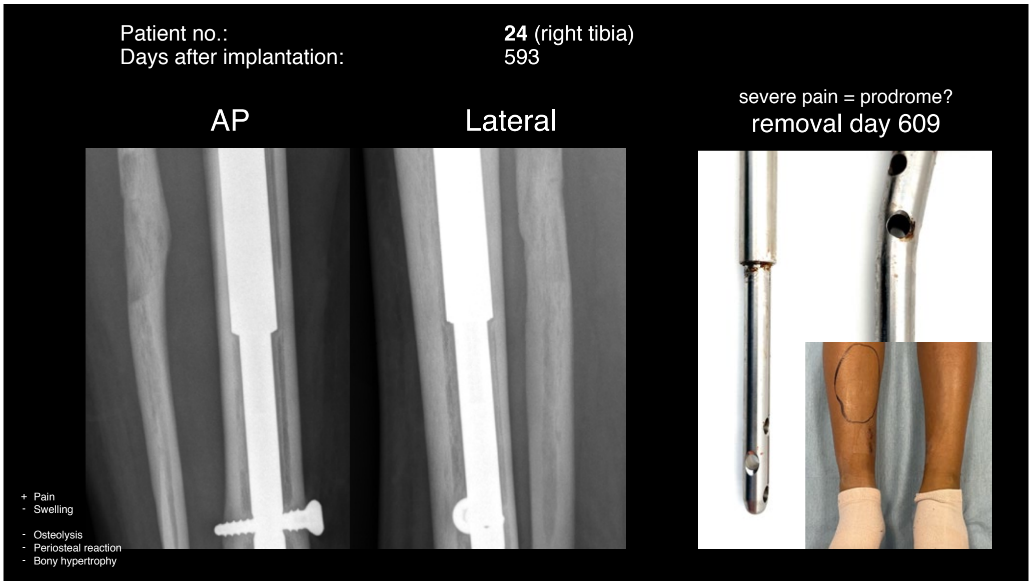
Patient no.: 24 (right tibia)
Days after implantation: 593
AP
Lateral
severe pain = prodrome?
removal day 609
+ Pain
- Swelling
- Osteolysis
- Periosteal reaction
- Bony hypertrophy

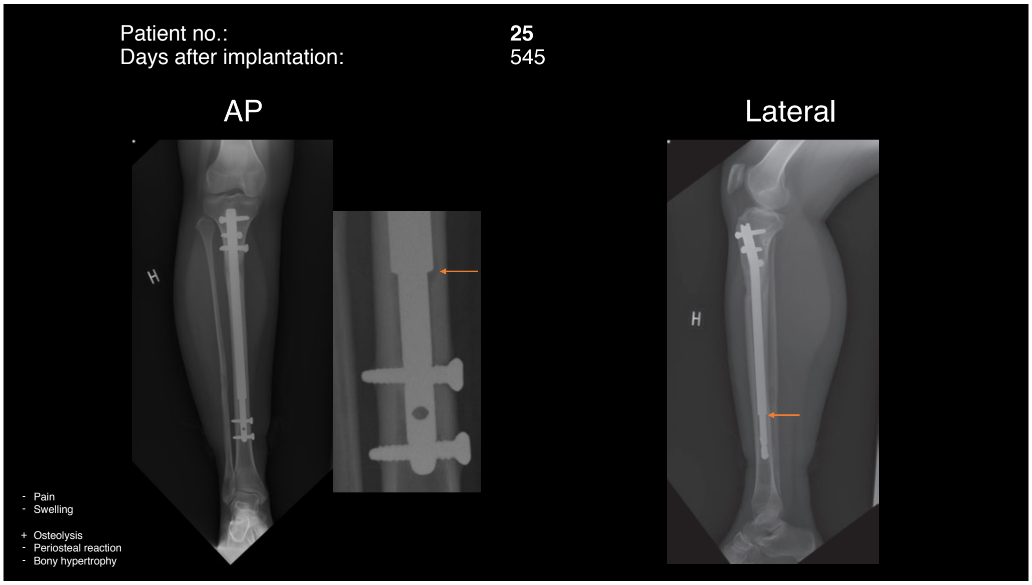
Patient no.: **25**
Days after implantation: 545
AP
Lateral
- Pain
- Swelling
+ Osteolysis
- Periosteal reaction
- Bony hypertrophy

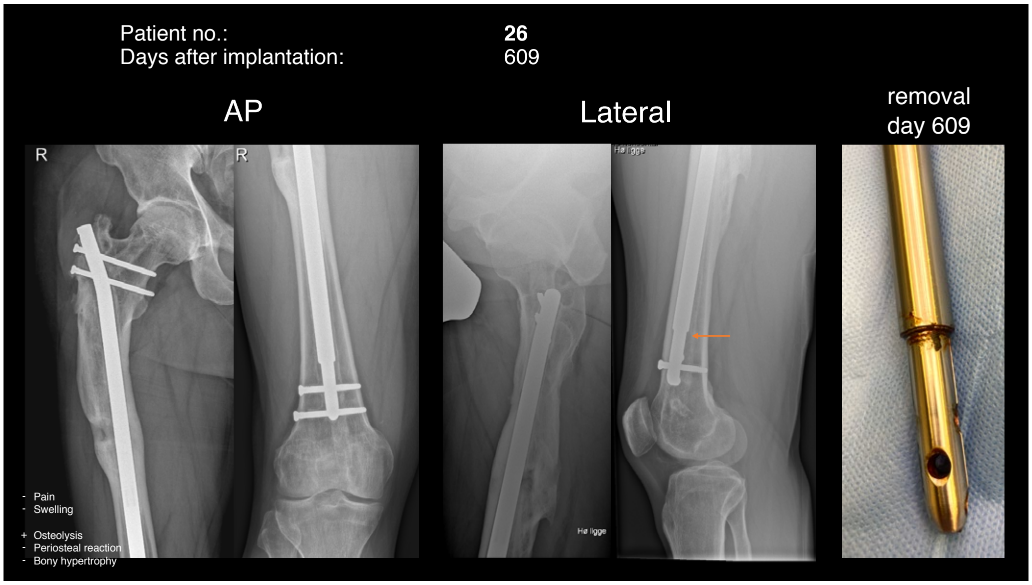
Patient no.: **26**
Days after implantation: 609
AP
Lateral
removal day 609
R
R
- Pain
- Swelling
+ Osteolysis
- Periosteal reaction
- Bony hypertrophy

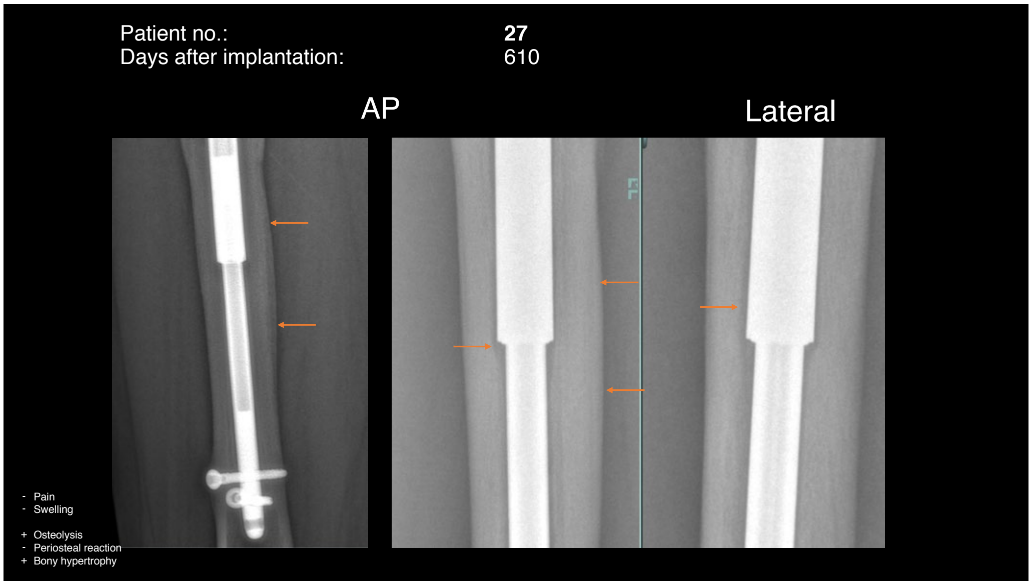
Patient no.: **27**
Days after implantation: 610
AP
Lateral
- Pain
- Swelling
+ Osteolysis
- Periosteal reaction
+ Bony hypertrophy
